# The Impact of Early-Life Cecal Microbiota Transplantation on Social Stress and Injurious Behaviors in Egg-Laying Chickens

**DOI:** 10.3390/microorganisms12030471

**Published:** 2024-02-26

**Authors:** Yuechi Fu, Jiaying Hu, Huanmin Zhang, Marisa A. Erasmus, Timothy A. Johnson, Heng-Wei Cheng

**Affiliations:** 1 Department of Animal Sciences, Purdue University, West Lafayette, IN 47907, USA; fu263@purdue.edu (Y.F.); hu165@purdue.edu (J.H.); merasmus@purdue.edu (M.A.E.); john2185@purdue.edu (T.A.J.); 2 U.S. National Poultry Research Center, USDA-ARS, Athens, GA 30605, USA; huanmin.zhang@usda.gov; 3 Livestock Behavior Research Unit, USDA-ARS, West Lafayette, IN 47907, USA

**Keywords:** aggression, cecal microbiota transplantation, gut microbiota, injurious behavior, laying hen, social stress

## Abstract

**Simple Summary:**

A plethora of studies have evidenced that the gut microbiota profoundly influences host brain function and behavioral characteristics in humans and various animals. In laying hens, it has been reported that injurious behaviors (such as aggressive pecking, feather pecking, and cannibalism) are associated with dysregulation of the microbiota–gut–brain axis. This study further investigated the effects of the early-life transplantation of different cecal contents on aggressiveness and related behaviors in chickens. Cecal bacterial profiles of two divergently selected inbred genetic lines (donors) were analyzed and then orally transferred separately into newly hatched male chicks of a commercial layer strain (recipients). Effects of cecal microbiota transplantation on growth, physiology, and behavior were examined in the recipient chicks. This study first evidenced that social stress and stress-related injurious behaviors in chickens can be reduced by modification of the gut microbiota composition and brain serotonergic activities via the gut–brain axis. The results provide new insights into understanding the cellular mechanisms of the gut microbiota in regulating stress-induced abnormal behaviors and offer a novel strategy for improving health and welfare in laying hens.

**Abstract:**

Injurious behaviors (i.e., aggressive pecking, feather pecking, and cannibalism) in laying hens are a critical issue facing the egg industry due to increased social stress and related health and welfare issues as well as economic losses. In humans, stress-induced dysbiosis increases gut permeability, releasing various neuroactive factors, causing neuroinflammation and related neuropsychiatric disorders via the microbiota–gut–brain axis, and consequently increasing the frequency and intensity of aggression and violent behaviors. Restoration of the imbalanced gut microbial composition has become a novel treatment strategy for mental illnesses, such as depression, anxiety, bipolar disorder, schizophrenia, impulsivity, and compulsivity. A similar function of modulating gut microbial composition following stress challenge may be present in egg-laying chickens. The avian cecum, as a multi-purpose organ, has the greatest bacterial biodiversity (bacterial diversity, richness, and species composition) along the gastrointestinal tract, with vitally important functions in maintaining physiological and behavioral homeostasis, especially during the periods of stress. To identify the effects of the gut microbiome on injurious behaviors in egg-laying chickens, we have designed and tested the effects of transferring cecal contents from two divergently selected inbred chicken lines on social stress and stress-related injurious behaviors in recipient chicks of a commercial layer strain. This article reports the outcomes from a multi-year study on the modification of gut microbiota composition to reduce injurious behaviors in egg-laying chickens. An important discovery of this corpus of experiments is that injurious behaviors in chickens can be reduced or inhibited through modifying the gut microbiota composition and brain serotonergic activities via the gut–brain axis, without donor-recipient genetic effects.

## 1. Introduction

Domestic egg-laying chickens have been continuously selected for high egg production with a high feed efficiency to meet the constant increase in human nutrition demand for eggs due to both population growth and rising individual consumption [1,2]. However, extreme selection is often at the expense of the animal’s health and welfare [3,4]; i.e., selecting one trait (such as production) could affect other traits, causing negative impacts on the animals [5]. Based on the traditional selection theory, an animal’s productivity is correlated with its competitive ability [6,7]. As unexpected results, the effects of selection for increased production reportedly resulted in increased interspecific competition and aggression [8,9,10]. In one of our previous studies, egg production increased significantly in former commercial Dekalb XL hens through more than 20 years of selection, while mortality associated with aggression and related injurious behaviors (aggressive pecking, severe feather pecking (SFP), and cannibalism) in non-beak trimmed hens also increased about 10-fold [11]. Increased injurious behaviors could be related to selection unequally affecting the animals’ adaptability to their rearing environments and management practices. Within a socioecological environment, not all animal individuals have an equal ability to modify their physiological and behavioral characteristics (such as personality traits for boldness, activity, and aggressiveness) in response to practice-associated stressors (inter-individual differences in adaption) [12,13,14,15]. Based on a dominance hierarchy or a ranking order, subordinates that are in direct contention with a dominant individual within a social group (i.e., the interactions between dominant higher-ranking (alpha) animals and submissive lower-ranking (beta) animals) exhibit fear, reducing their adaptation to the rearing environments and related management practices. Consequently, the subordinates enter a ‘pre-pathological state’ or ‘pathological state’ with physiological and metabolic disturbances [16,17,18]. Dominant chickens then exhibit an increased frequency of aggression and related injurious behaviors via the brain award systems and reinforced learning pathways, which could be similar to the brain systems reported in humans [19]. 

Aggression in chickens, as in most other species of social animals, is a highly complex social behavior. From an evolutionary viewpoint, aggression, as a natural part of an animal’s life, is essential for the animal to establish and maintain social status, to protect valuable resources (food and territory), and to reproduce successfully (survival, growth, breeding, and rearing offspring) [20,21,22,23]. However, some forms of aggression in chickens, such as excessive aggression-related injurious behaviors, can be harmful, leading to devastating consequences with increased social stress, feather and body damage, and injury (leading to cannibalism) [24,25,26]. In addition, numerous studies focusing on the function of gut microbiota in behavioral development have indicated that the dysregulation of the microbiota–gut–brain (MGB) axis has been implicated in abnormal behaviors (aggressive pecking, feather pecking (FP), and cannibalism) in laying hens [27,28]. Feather pecking may not be associated with dominance status; however, recent studies suggested that FP is related to social-stress-associated fearfulness [29]. Injurious behaviors, as a socially transmitted learning behavior, can be spread among flocks [30]. It has been previously reported that FP could affect up to 80% of birds in current housing environments [25].

Those injurious behaviors may be reduced through genetic selection [31,32,33,34]. However, there is “no sign that breeders will be able to guarantee the ‘non-peck’ layers in time” for hens to be housed in cage-free systems [35,36]. Egg production facilities are transferring from the conventional (battery) cage system to cage-free systems in the United States. Approximately 230 corporate customers, such as McDonald’s, Walmart, Subway, and Kroger, have pledged to only buy cage-free eggs by or before 2025. In addition, recent studies showed that selection for low-FP chickens failed to eliminate FP completely in flocks [37], which suggests that genetic selection should be paired with other management strategies [38]. Currently, beak trimming (BT), a routine procedure practiced in the United States egg industry, is the most effective method for reducing social stress by preventing and/or inhibiting injurious behaviors. However, BT has been criticized for causing tissue damage and pain (acute, chronic, or both) [39,40], negatively impacting the welfare of billions of chickens annually [41,42]. In addition, the chicken beak is a multipurpose organ playing a vital role in a variety of functions, from eating to defense against predators and parasites [43]. Beak trimming damages these beak functions, leading to abnormal behaviors and frustration [44]. Considerable concerns from the public have led to a growing global movement against the procedures causing pain and suffering in farm animals. However, recent studies have reported that FP and cannibalism still occur in beak-trimmed, floor-reared, and cage-free flocks [45]. Based on the outcomes, several studies have advocated that “solutions have to be found before thinking about banning BT” [45,46]. In addition, recent studies have indicated that FP and foraging are uncorrelated, which is inconsistent with the original hypothesis that FP is redirected food-related foraging pecks [47]. Feather pecking can lead to cannibalistic pecking, consequently eating and removing flesh from the victims by further reinforcing the behavior via the gut–brain reward systems (the central serotonergic and dopaminergic systems) [48,49]. In addition, injurious-behavior-associated social stress can disturb intestinal bacterial balance, resulting in physiological and behavioral disorders via the MGB axis [50,51].

The gut microbiota plays a critical role in early programming and later activity of the central stress systems, i.e., the hypothalamic–pituitary–adrenal (HPA) and the sympathetic–adrenal–medullary (SAM) axes [52,53,54]. Like an endocrine organ, the gut microbiota is sensitive and reactive to various exogenous stimuli, functioning as an environmental sensor linked to the pathogenesis of stress-related illnesses through the bidirectional communication of the MGB axis [55,56,57,58,59] and the microbiota–gut–immune (MGI) axis [60,61] in various animals including chickens [62,63,64]. Maintaining gut microbiota balance and health is essential for animals (including chickens) to maintain their optimal physiological and behavioral functions of growth, reproduction, and welfare. In humans, various psychological (emotional and mental overstimulation) and/or physical (environmental conditions) stressors alter gut microbiota diversity, composition, or both and increase the inability to maintain a healthy gut microbial profile, leading to neuropsychiatric disorders [65,66,67,68,69,70,71]. Targeting the intestinal microbiota with the goal of restoring its balance has been recognized as a novel therapeutic option for patients with neuropsychiatric disorders [72,73,74]. Several probiotics, as psychobiotics, such as *Bifidobacterium* and *Lactobacillus*, which can benefit mental health, have been used for preventing and treating patients with behavioral impairment, such as anxiety, depression, and impulsively and compulsively disrupted social behavior, via regulating the MGB, MGI, or both axes [75,76,77,78,79,80,81,82,83,84]. However, the use of purified probiotics benefits has shown mixed results, with several weaknesses including transient beneficial effects, requiring continuous administration over time due to the host’s resident microbial populations and “colonization resistance” [85]. Thus, it has been proposed that using live commensals coming directly from a healthy gut may be more effective than probiotics [86,87]. However, this hypothesis has not been well investigated in chickens.

The avian cecum, as a multipurpose organ, has a greater biological role than the cecum in most mammals [88,89,90,91]. In addition, chicken lines’ differences in the cecal microbiota composition in response to environmental stressors (such as ambient stress) [92] and experimental challenge models [93] have been reported. For example, laying hens showing high or low FP have different gut microbial populations [94,95] and intestinal and peripheral metabolite profiles [96,97]. However, a recent study reported that these differences may not be associated with FP and antagonistic behavior, due to limited effects on microbiota composition between the divergently selected lines for high and low FP [98]. It is still unclear how the gut microbiota is involved in injurious behaviors. In addition, the effects of early-life microbiota transplantation on gut microbiota composition and its function have not been well established [99]. For these reasons, we have designed and tested our hypothesis: modulation of the gut microbiota via cecal microbiota transplantation (CMT) from divergently selected inbred genetic lines (donors) would alter injurious behaviors in egg-laying chickens (recipients).

## 2. Genetic Lines and Study Design

In our pilot study, chicks (day-old) orally inoculated with cecal microbiota from divergently selected donors (non-aggressive or aggressive hens) altered injurious behaviors in recipients; i.e., non-aggressive donors’ recipients showed less aggressive pecking than aggressive donors’ recipients with higher brain serotonergic activities.

### 2.1. Unique Production, Biology, and Behavior between the Divergently Selected Inbred Lines

Two unique highly inbred white leghorn chicken lines have been continuously selected for resistance (line 6_3_) or susceptibility (line 7_2_) to Marek’s disease since the late 1960s [100,101,102]. This selection leads to line differences in production performance [103], neuroendocrine function [104,105,106], immunity [107,108,109,110], and behavior [111,112]. Compared to line 6_3_ chickens, line 7_2_ chickens have a higher number of CD4+ T cells but a lower number of CD8+ T cells [113,114] with suppressed cellular immunity [115]. In addition, the expression of cytokine (interleukin (IL)-6 and IL-18) mRNA in response to Marek’s disease virus infection is significantly different between the two inbred lines, of which line 7_2_ chickens express higher levels of both cytokines than line 6_3_ chickens [116], while line 6_3_ chickens have higher gene expressions of toll-like receptor (TLR)-3, TLR-7, and IL-8 [117]. Toll-like receptors, as a class of proteins, are expressed on the membranes of various immune cells, playing a key role in the innate immune system. IL-8, as a chemoattractant cytokine, attracts and activates neutrophils in inflammatory regions via regulating the innate immune system. In addition, line differences in social stress and stress-induced aggressive behaviors have been observed; line 7_2_ chickens have higher heterophil-to-lymphocyte (H/L) ratios (a stress indicator) with more aggressive pecks and longer durations of fights than those of line 6_3_ chickens [102,104,112]. The differences in behaviors could be related to the line differences in serotonergic activities [105]. Line 7_2_ chickens have lower levels of brain serotonin (5-HT) than line 6_3_ chickens. Serotonin dysregulation has been implicated in a range of neuropsychiatric disorders in humans and various animals including chickens [118,119]. Lower levels of 5-HT have also been found in the brain of violent offenders [120,121,122]. The unique divergently selected inbred lines provide useful models for investigating gut microbiota effects on injurious behaviors in chickens. To understand the role of the cecal microbiome in regulating injurious behaviors, the following trials were conducted using cecal contents from the two inbred chicken lines.

### 2.2. Study Design and Results

#### 2.2.1. Trial 1 [112]

The aim of this trial was to determine the correlations between aggressive behavior, gut microbiota, and physiological characteristics of the divergently selected laying hens (lines 6_3_ vs. 7_2_). The samples of blood, brain (the raphe nucleus), and cecal content were collected from ten sixty-week-old hens per line (*n* = 10). Monoamines of the raphe nucleus (serotonin, 5-HT; 5-hydroxyindoleacetic acid, 5-HIAA; tryptophan, TRP; epinephrine, EP; and norepinephrine, NE) were measured using high-performance liquid chromatography (HPLC). Peripheral (plasma) 5-HT and TRP, cytokines (IL-2, IL-6, IL-10, and tumor necrosis factor, TNF-α), and immunoglobulin (Ig) G were detected in duplicate using enzyme-linked immunosorbent assay (ELISA). Plasma corticosterone (CORT) concentration was measured in duplicate using radioimmunoassay (RIA). The number of peripheral white blood cells was measured and then the H/L ratio was calculated. Cecal contents were used for determining the line differences of the microbiota composition using 16S rRNA sequencing analysis, and functional predictions were performed.

**Figure 1:**
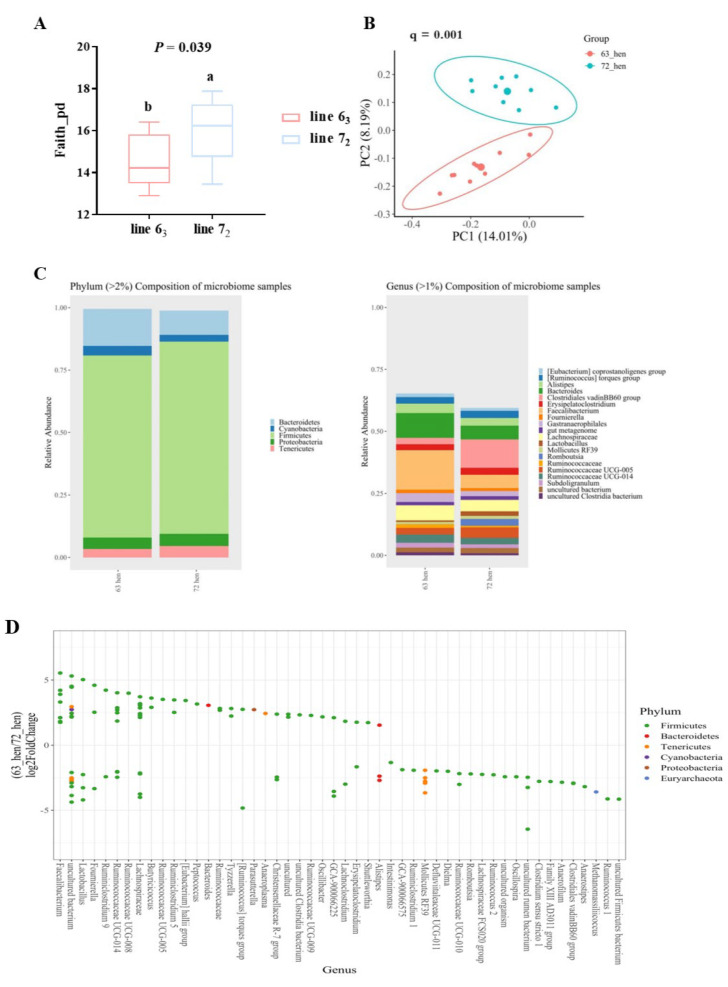
Microbiota profile between two diversely selected chicken lines 6_3_ and 7_2_ (*n* = 10). (**A**) Faith’s PD index, values are median ± SEM, ^a,b^ indicates significant differences (*p* ≤ 0.05). (**B**) Principal coordinate analysis (PCoA) of Bray–Curtis similarity. Each dot represents one bird (*n* = 10), and PCo1 and PCo2 represent the percentage of variance explained by each coordinate. (**C**) Cecal microbial composition profiles of the recipient chickens at phylum and genus (relative abundance >2% at phylum, >1% at genus) levels. (**D**) DESeq2 analysis of differentially abundant ASVs between line 6_3_ and line 7_2_. Estimations of log2 fold change values for each ASV were computed and each point represents an ASV that was significantly different (*p* ≤ 0.05) [106].

**Figure 2:**
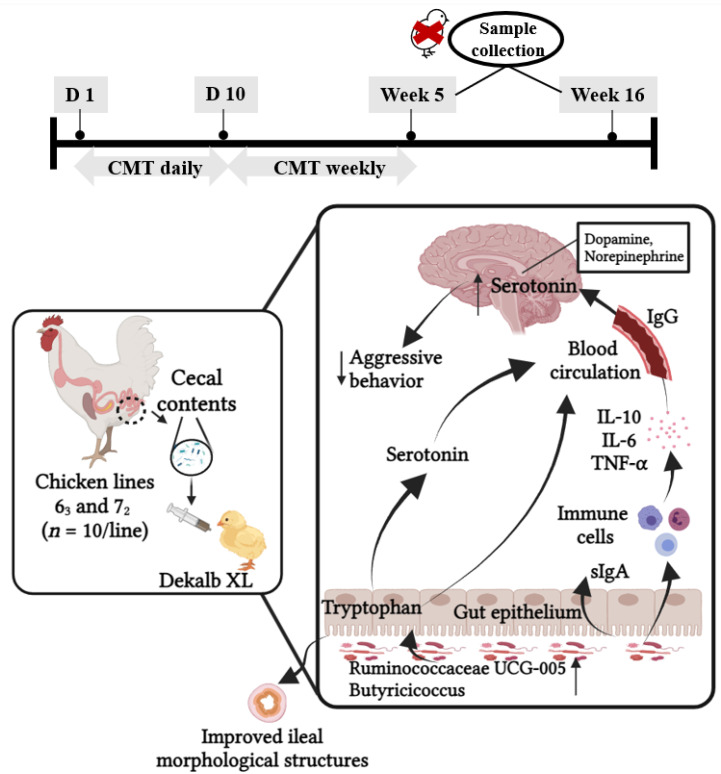
Timeline of the experimental design of trial 2 and trial 3 and the proposed mechanisms underlying the transplant effects on health and behavior of recipient chickens.

The results showed that central 5-HT and TRP levels were higher in line 6_3_ chickens compared to those of line 7_2_ chickens (*p* < 0.05, Table 1A). In addition, both CORT concentrations and H/L ratios were lower in line 6_3_ chickens (*p* < 0.05, Table 1B). The level of TNF-α tended to be higher in line 6_3_ chickens (*p* = 0.09, Table 1C). Line differences in the cecal microbial community were also found between line 6_3_ and line 7_2_ chickens. Line 7_2_ chickens had higher phylogenetic diversity than line 6_3_ chickens, with distinct microbiota composition differences (Figure 1A,B). *Faecalibacterium, Oscillibacter, Butyricicoccus,* and *Bacteriodes* were enriched in line 6_3_ chickens, while *Clostridiales vadin BB60, Alistipes,* and *Mollicutes RF39* were dominant in line 7_2_ chickens (Figure 1C,D). Like the previous findings [105], function prediction from PICRUSt2 indicated that the kynurenine pathway (KP) was enriched in line 7_2_ chickens, while tryptophan–serotonergic activity was inherently higher in line 6_3_ chickens. The KP of tryptophan metabolism (degraded more than 90% of absorbed dietary TRP) plays a critical role in psychiatric disorders as many kynurenine metabolites are neuroactive factors modulating neuroplasticity and/or exerting neurotoxic effects. These results suggest there is a functional linkage between the line differences in the serotonergic activity, stress response, innate immunity, and cecal microbiota populations, which provides a rationale of the hypothesis that microbiota transplantation at an early age may be a novel strategy for reducing the stress response and stress-related injurious behaviors in chickens. Based on the outcomes, trials 2 and 3 were designed and conducted (Figure 2).

#### 2.2.2. Trial 2 [106]

The aim of this trial was to determine the effects of early-life CMT from the divergently selected inbred lines on growth, gut 5-HT, and immunity in recipient chickens. The cecal contents were randomly collected from 10 sixty-week-old hens per inbred line (donors). The collected samples were evenly pooled within the line and then diluted 1:10 with gut microbiome media. The recipients were a commercial strain, Dekalb-XL-line chickens. The oral gavage of diluted cecal microbiota was conducted once daily from day 1 to day 10, and then boosted once weekly from week 3 to week 5. Eighty-four 1-day-old male chicks were randomly assigned to 3 treatments with 7 cages and 4 chicks per cage for a 16-week trial (*n* = 7): CTRL (control, 0.1 mL NaCl saline), 6_3_-CMT (0.1 mL cecal solution of line 6_3_), and 7_2_-CMT (0.1 mL cecal solution of line 7_2_). The male chicks were used in this study as male chickens tend to be more aggressive than female chickens due to the hormonal differences. In weeks 5 and 16, the blood samples were collected for H/L ratios, and the levels of cytokines (IL-6, IL-10, and TNF-α), IgG, and CORT were measured using white blood cell counting, ELISA, and RIA, respectively. The spleen samples were used for mRNA expression of cytokines by RT-qPCR, and the ileal samples—two 3.5 cm sections (near the diverticulum)—were collected for histomorphological analysis using a routine hematoxylin and eosin procedure. Gut serotonergic activity (TRP, 5-HT, 5-HIAA, and 5-HIAA/5-HT ratio) and secretory (s) IgA were analyzed using HPLC and ELISA, respectively. The body weight was also collected for calculating the relative weight of the adrenal gland. 

**Figure 3:**
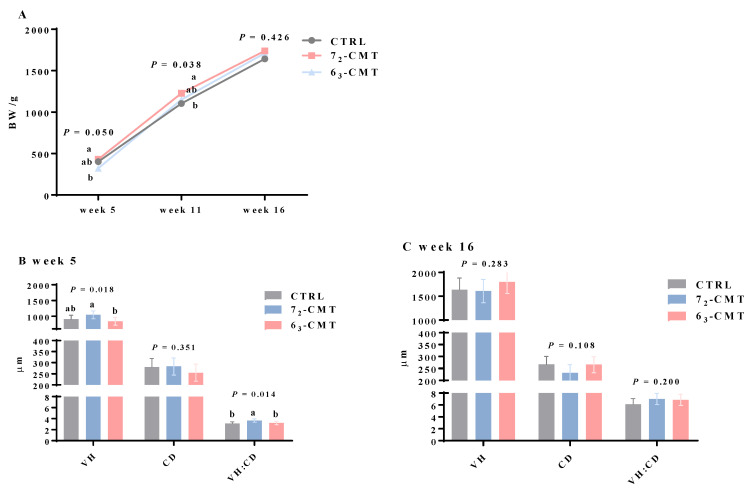
Effects of cecal microbiota transplantation on (**A**) body weight of recipient roosters; ileal morphology of recipient roosters in week 5 (**B**) and week 16 (**C**). Ileal villus height (VH), crypt depth (CD), and VH/CD ratio. Values are least-squares means ± SEM, *n* = 7. ^a,b^ indicate significant differences (*p* ≤ 0.05). Abbreviations: 6_3_-CMT, chickens with cecal bacterial solution of donor line 6_3_; 7_2_-CMT, chickens with cecal bacterial solution of donor line 7_2_; CTRL, control; CD, crypt depth; VH, villus height [106].

**Figure 4:**
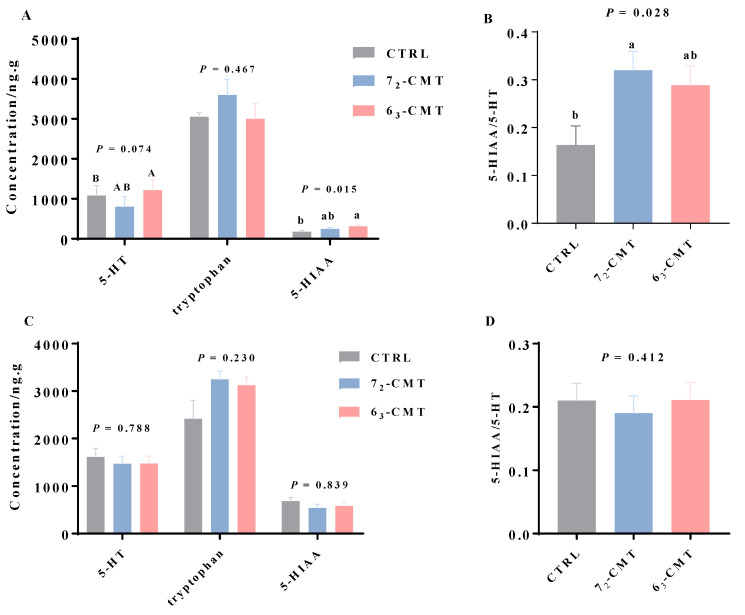
Effects of cecal microbiota transplantation on ileal serotonergic activities of recipient roosters. Serotonergic activity in week 5 (**A**,**B**) and week 16 (**C**,**D**). Values are least-squares means ± SEM, *n* = 7. ^a,b^ indicate significant differences (*P* ≤ 0.05), ^A,B^ show trend differences (0.05 < *P* ≤ 0.10). Abbreviations: 5-HIAA, 5-hydroxuindoleacetic acid; 5-HT, serotonin; 6_3_-CMT, chickens with cecal bacterial solution of donor line 6_3_; 7_2_-CMT, chickens with cecal bacterial solution of donor line 7_2_; CTRL, control [106].

The results showed that compared to 7_2_-CMT chickens, 6_3_-CMT chickens had a lower body weight and ileal villus/crypt ratio among the treatments in week 5 (Figure 3 and Figure S1). In addition, 6_3_-CMT chickens had an improved stress adaptive capacity: lower H/L ratios, together with a tendency of a lower relative adrenal gland weight in week 16 (Table 2). 6_3_-CMT chickens also had higher plasma levels of IL-10, with lower levels of plasma natural IgG, with a tendency of lower levels of IL-6 in week 16 (Table 3). In contrast, 7_2_-CMT chickens had a lower concentration of ileal mucosal sIgA in week 5 with a tendency for a higher mRNA abundance of splenic IL-6 and TNF-α in week 16 (Table 4). Furthermore, 6_3_-CMT chickens tended to have the highest 5-HT concentrations with the highest serotonergic turnover in the ileum in week 5 (Figure 4). These results indicate that early-postnatal CMT from the different donors (lines) was associated with the different patterns of growth and health status through regulating the ileal morphological structures, gut-derived serotonergic activity, peripheral cytokines, and antibody production, as well as stress responses in recipient chickens. The findings confirm our hypothesis that transferring cecal contents at an early age has unique line effects, including growth, immunity, and gut neurotransmitter synthesis with a long-lasting effect. The current findings may also indicate that the gut microbial function is without donor-recipient genetic effects (i.e., with the line’s unique biologic characteristics being transferred from the selected inbred donors to the third commercial recipient line regardless of the lines’ genetic backgrounds).

#### 2.2.3. Trial 3 [123]

The aim of this trial was to determine the effects of early-life CMT from the divergently selected inbred chicken lines (donors) on cecal microbiota profile, brain monoamines, aggression, and their correlations in recipient chickens. The samples of the brain (the hypothalamus) and cecal contents of recipients were collected. The monoamines (5-HT, EP, NE, and DA) of the hypothalamus were measured in triplicate using HPLC, and cecal samples were analyzed using 16S rRNA gene sequencing. The aggressive behaviors were observed using both home-cage video analysis and paired behavioral tests based on the previously developed definitions [124,125,126]. The paired test is a routine method used for analyzing aggression-related social ranking as well as fear and anxiety in chickens [127,128,129]. Its rationale and mechanisms are similar to the resident–intruder test used in rodents, a standardized test for detecting social-stress-induced aggression and related violence [130].

**Figure 5:**
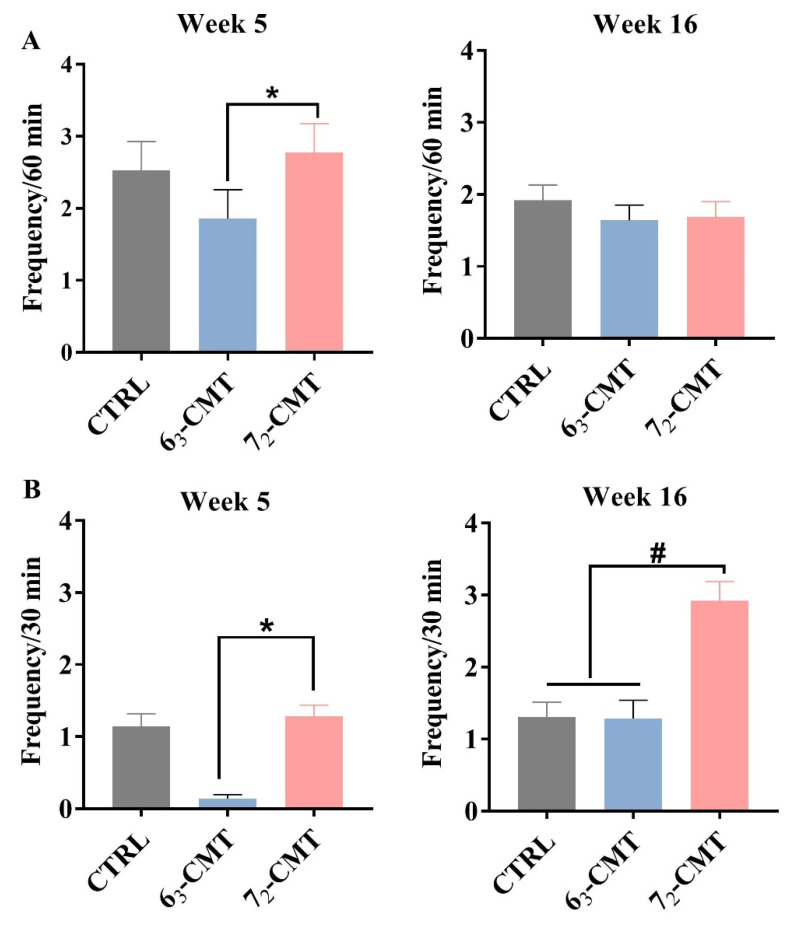
Frequency of aggressive pecking of recipient chickens in week 5 and week 16. (**A**) Home-cage behavior. (**B**) Paired test. Values are means ± SEM, *n* = 7. * indicates significant differences (*p* ≤ 0.05), and ^#^ shows trend differences (0.05 < *p* ≤ 0.1). 6_3_-CMT, received cecal content solution from 6_3_ donors; 7_2_-CMT, received cecal content solution from 7_2_ donors; CTRL, received saline, control [123].

**Figure 6:**
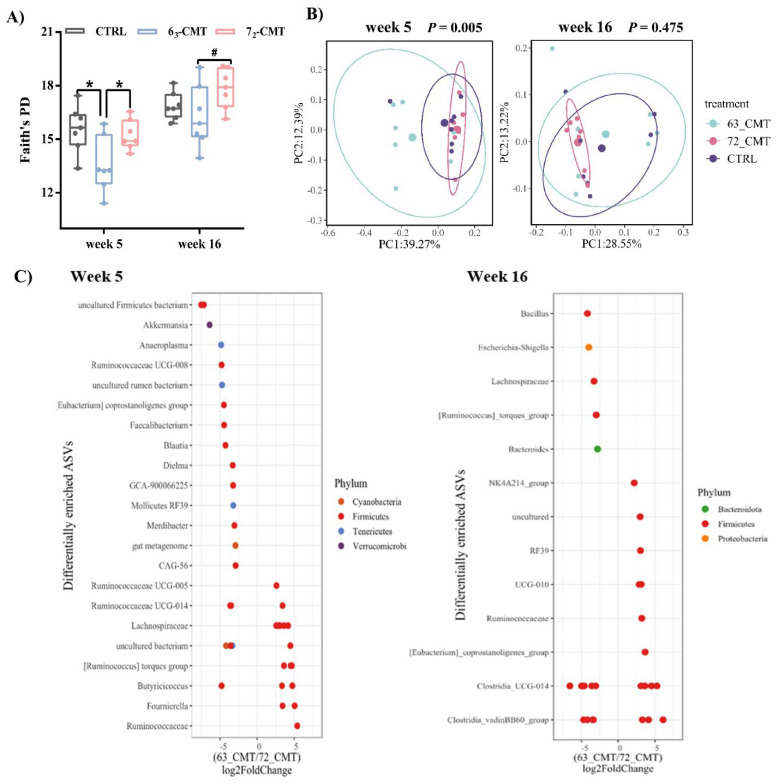
Effects of cecal microbiota transplantation on cecal microbial profiles of recipient chickens in week 5 and week 16 (*n* = 7). (**A**) Faith’s PD index, values are median ± SEM, * indicates significant differences (*p* ≤ 0.05), and ^#^ shows trend differences (0.05 < *p* ≤ 0.1). (**B**) Principal coordinate analysis (PCoA) of Unweighted UniFrac of recipient chickens in week 5 and week 16. Each dot represents one bird (*n* = 7), and PCo1 and PCo2 represent the percentage of variance explained by each coordinate. (**C**) DESeq2 analysis of differentially abundant ASVs between 6_3_-CMT group and 7_2_-CMT group in week 5 and week 16. Estimations of log2 fold change values for each ASV were computed and each point represents an ASV that was significantly different (*p* ≤ 0.05). 6_3_-CMT, received cecal content solution from 6_3_ donors; 7_2_-CMT, received cecal content solution from 7_2_ donors; CTRL, received saline, control [123].

Data indicated that compared to 7_2_-CMT recipients, 6_3_-CMT recipients showed less aggressive behaviors (Figure 5) with a higher serotonergic activity, evidenced by higher concentrations of 5-HT and 5-HIAA (Table 5) in the hypothalamus in week 5 with a tendency for higher concentrations of TRP in week 16. Tryptophan can pass the blood–brain barrier and is the sole precursor of 5-HT [51], which may indirectly indicate how to reduce aggressive behaviors in the recipient chickens via activating the brain serotonergic system. Through 16S rRNA gene sequence analysis, we observed that CMT-induced microbiota changes, that is, a distinct microbial community diversity, were observed between 6_3_-CMT and 7_2_-CMT recipients. 7_2_-CMT recipient chickens had a higher phylogenetic diversity than 6_3_-CMT recipient chickens in weeks 5 and 16 (Figure 6A). Cecal microbiota transplantation also induced changes in microbial community structures (Unweighted UniFrac) among treatments in week 5 but not in week 16 (Figure 6B). Compared to 6_3_-CMT chickens, 7_2_-CMT chickens had enriched ASVs belonging to 14 genera, including *Akkermansia, Anaeroplasma, Ruminococcaceae UCG-008, Faecalibacterium, Blautia, Dielma, GCA-900066225, Merdibacter,* and *CAG-56*, while ASVs belonging to 5 genera were more abundant in 6_3_-CMT recipients including *Ruminococcaceae UCG-005, Ruminococcaceae UCG-014, Lachnospiraceae,* and *Fournierella*. In week 16, compared to 6_3_-CMT recipients, ASVs belonging to 5 genera (including *Bacillus, Escherichia-Shigella, Lachnospiraceae,* and *Bacteroides*) were more abundant in the 7_2_-CMT recipients, while ASVs belonging to 6 genera were more abundant in 6_3_-CMT recipients (including *Ruminococcaceae NK4A214 group, Ruminococcaceae,* and *Eubacterium coprostanoligenes* group) (Figure 6C). The results suggest that CMT at an early age affects the development of the gut microbiota composition and reduces aggressive behaviors in recipient chickens via regulating the activities of the brain serotonergic system through the gut–brain axis.

## 3. Cecal Microbiota Transplantation, Social Stress, and Injurious Behavior in Chickens

### 3.1. Stress and Gut Microbiota

Stress is a natural biological (physical and mental) response to internal and external challenges in living organisms, including chickens. Normally, it prompts chickens’ ability to adapt to their rearing environments, while abnormally, an overload of stress challenge (too much exposure to a stressor or combined stressors causing a long-term activation of the stress response systems) reduces gut microbiota diversity, composition, or both [131]. The gut microbiota is functionally like an endocrine organ, releasing numerous bioactive factors to activate the HPA and SAM stress systems in response to stimulations, consequently affecting host physiological and behavioral homeostasis via the bidirectional communication of the MGB and MGI axes [63,64]. Healthy intestinal microbial communities and functions are essential for animals to fit their living environments [132,133]. The intestinal microbial community has been named the “social or behavioral immune system” linked to the microbiota–gut–brain–immune axis [134] based on the two reciprocal themes: (1) that gut microbiota influences host social behavior and (2) that social behavior and social structure shape the composition of the gut microbiota across individuals [135]. Based on these theories, environmental factors causing changes in the gut microbiome are linked to stress-induced neurobehavioral disorders including aggression and related damaging behaviors [136,137]. In addition, the differences in gut microbiota composition and/or diversity are related to personality traits [15,138], temperament [139,140], and sociability [87,141] in humans and various social animals, including chickens.

Numerous psychological (an emotion and/or mental overstimulation) and/or physical (environmental conditions) stressors reduce gut microbiota diversity and/or alter microbiome composition by (1) disrupting the community stability of commensal bacterial populations, often accompanied by reduced beneficial bacteria and increased pathogens (causing a chronic low-grade inflammation); (2) increasing the survival translocation of pathogens and releasing virulence factors; (3) disrupting absorption of nutrients and minerals (metabolic disorders); (4) disrupting microbial neuroendocrine functions (alterations in synthesis of several signaling molecules and neurochemicals including 5-HT in the GIT); (5) disrupting the gut epithelial barrier, thereby increasing intestinal permeability and releasing certain bacteria, bacterial antigens, and metabolites (leaky gut), resulting in both intestinal and systemic immune reactions; and (6) damaging epithelial cells, producing free radicals and reducing antioxidant capacity (oxidative stress) [142,143,144]. These changes in the gut microbiota with a chronic low-grade inflammation profoundly influence host health and behavioral homeostasis via the MGB and MGI axes [58,145]. Treatments aimed at restoring normal gut microbiota composition and homeostasis have become effective methods to prevent and/or reduce various stress-induced neuropsychiatric disorders [146,147].

### 3.2. Possible Pathophysiological Mechanisms Underlying Injurious Behaviors in Chickens

In mammals, chronic stress is a major risk factor in neuropsychiatric disorders [148]. Social stress induces numerous microbiota-derived neurochemicals (neuromodulators) to enter the blood stream and influence brain function, especially the functions of both the HPA and SAM axes [149,150], which affects the development and balance of emotional and mental behaviors. Alterations in neuroendocrine homeostasis, i.e., CORT and catecholamines (such as EP and NE) levels, have been identified as the final common pathways in controlling animal behavior and pathophysiological status [151]. Animals raised in a germ-free (GF) environment expressing an exaggerated HPA response to psychological stressors could be normalized with certain bacterial probiotic species, such as *Bifidobacterium infantis* [152,153] and *Bacillus licheniformis* [154]. The animals treated with probiotics had a blunted HPA response [155]. Similarly, FP in chickens is influenced by dysregulation of the gut microbiome, which consequently affects neurotransmitter and immune homeostasis [27,94,95]. Current studies have evidenced that changing prenatal and early postnatal brain developments are involved in the development of injurious behaviors in laying hens [156] and other farm animals [157]. Our current studies have evidenced that early-life CMT induced different levels of aggressive behavior in the male recipients, which is corrected with each donor line’s behaviors. The results indicate that transferred donors’ cecal microbiota uniquely modifies the serotonergic activity, stress response, innate immunity, and cecal microbiota populations in recipients through the MGB and MGI axes. The underlying mechanisms, such as the responsible individual bacterium (or bacteria), the released neuromodulators and/or metabolites, as well as the involved pathways, will be examined in upcoming studies.

### 3.3. Physiological Mechanisms of Modulation of Intestinal Microbiota to Regulate Social Stress and Related Abnormal Behaviors

A healthy intestinal microbial community plays a critical role in regulating stress responses of the HPA and SAM axes to maintain host behavioral and physiological functions to fit their living environments [132]. Accumulating studies from various animal models in gut microbiota investigations, such as GF (complete absence of microbial exposure) animals, SPF (specific pathogen-free) animals, antibiotic-treated (broad-spectrum antibiotic cocktails) animals, and animals exposed to pathogenic bacterial infections, suggest that the gut microbiota plays an important role in the regulation of anxiety, mood, and cognition, indicating the possibility of using probiotics to modify the gut microbiota to control impulsive and compulsive behaviors in patients with neuropsychiatric disorders [158,159,160,161]. Like mammals, the gut microbiome plays a critical role in poultry health and welfare [119,162,163]. Laying hens showing high or low FP have different gut microbial populations [27,94,95,164] and metabolite profiles [96,97]. Therefore, the gut microbiome represents a novel therapeutic target for stress-induced mental and mood disorders in humans and injurious behaviors in chickens.

Probiotics are commensal bacteria that offer potential health benefits to the host, including the allostatic load (cumulating chronic stress effects on the body), when administered in adequate amounts. Generally, probiotics may aid animals in adapting to their ambient environments and protect against pathogens by (1) altering the microbiota profile in favor of beneficial bacteria to prevent the growth of pathogens and compete with enteric pathogens for the limited availability of nutrient and attachment sites; (2) producing bacteriocins (including bacteriostatic and bactericidal substances) and short-chain fatty acids against pathogens to regulate the activity of intestinal digestive enzymes and energy homeostasis and increase mineral solubility; (3) modulating host immune and inflammatory responses to reduce oxidative stress, inflammation, and cell injury; (4) restoring/strengthening the intestinal barrier integrity, which prevents pathogens and toxic substances from crossing the mucosal epithelium; (5) stimulating the neuroendocrine system and attenuating stress-induced disorders of the HPA and/or SAM axes via the MGB and MGI axes; and/or (6) inducing epithelial heat shock proteins to protect cells from oxidative damage [165,166,167,168,169,170]. Both human and rodent studies indicated that probiotics reduce chronic-psychological-stress-induced abnormal brain activity and related cognitive dysfunctions by lowering plasma CORT and adrenocorticotropic hormone levels, restoring hippocampal 5-HT and NE levels, and normalizing immunity with low plasma levels of TNF-α but high levels of IL-10, an anti-inflammatory cytokine [171,172,173]. Several probiotics, as psychobiotics, for example, *Bifidobacterium* and *Lactobacillus*, deliver mental health benefits with neurobehavioral effects, which have been used in humans for improving cognitive function and for preventing and treating patients with behavioral impairment in neurodegenerative diseases, such as Alzheimer’s disease and Parkinson’s disease, and in diseases with neuropsychiatric disorders, such as anxiety, depression, and impulsively and compulsively disrupted social behavior [75,77,78,79,83,84]. Based on findings, targeting the gut microbiota has been recognized as a novel therapeutic option for patients with neuropsychiatric disorders [63,73,74]. Current studies have evidenced that the influence of the gut microbiota on the host behavior as seen in mammals is shared in chickens [173]. For example, dietary supplements of probiotics-based *Bacillus amyloliquefaciens* reduce distress calls and aggressive behavior in turkey poults [174], and *Lactobacillus rhamnosus* [175,176] and *Bacillus subtilis* [177] decrease stress-induced FP in adult hens by restoring the gut microbiota and 5-HT metabolism [70]. However, the evidence for probiotic benefits is mixed, proposing that the use of live commensals coming directly from a healthy gut may be more effective than probiotics.

Fecal microbiota transplantation has recently become a novel method for modulating the gut microbiota in gastrointestinal disorders such as inflammatory bowel syndrome and CDI [178,179], and non-gastrointestinal diseases including neuropsychiatric disorders [180,181]. Fecal microbiota transplantation is a method of directly restoring healthy gut bacteria by transferring stool from a healthy donor. Stool contains thousands of microorganisms and a vast number of metabolites and has been recognized as a rapid and effective method to reshape the intestinal microbiota and metabolic profiles in humans and animals [182,183]. For example, the gut microbiota of recipients from stressed donors mimics the effects of stress on control animals, which could be reversed by transferring microbiota from unstressed animals [184,185]. Studies in CDI patients revealed that the diversity of gut microbiota is increased following FMT, which is critical for defense against pathogens via colonization resistance. Clinically, a single dose can have long-lasting effects [186,187,188]. However, recent studies indicate that a fecal sample is not reliable in mapping the complete cecal microbiome and cannot be used to monitor the shifts and changes in cecal content in chickens [189,190,191].

Taken together, in humans and rodents, microbial colonization impacts brain development in early life, with long-lasting effects on adult behavior. Fecal microbiota transplantation and probiotics repair the social-stress-induced disturbance of microbial functions and attenuate the stress-induced responses of the HPA and/or SAM axes by protecting neuronal plasticity at the hypothalamic level as well as promoting neurogenesis in the hippocampus. Fecal microbiota transplantation restores the negative feedback of the stress systems to regulate animal health and behavior, providing novel insights into understanding how the gut microbiota community prevents abnormal behavior in patients with psychological disorders. We hypothesized that similar cellular mechanisms may be manifested in CMT recipient chickens, because chickens and mammals share a similarity in the interactions between the microbiome and the neuroendocrine systems, generally named microbial endocrinology [192,193,194]. This hypothesis has been tested and evidenced in our recent studies.

### 3.4. Cecal Microbiota Transplantation and Injurious Behavior in Chickens 

Early life (immediate post-hatch) in chickens is a critical window of time causing enduring effects on the development of the intestinal microbiome and related brain functions and behavior in later life. Although microbial complexity considerably increases in the cecum with age [157], modulation of the structure and function of the cecal microbiome during early life alters neurophysiology in adolescence [195]. In chickens, the avian cecum plays a vitally important role in maintaining pathophysiological homeostasis, especially during periods of stress [196,197,198,199]. With up to 10^11^ cells per gram of content, the cecum has the greatest bacterial biodiversity (bacterial diversity, richness, and species composition) along the chicken GIT [200,201,202]. As a multi-purpose organ, it has a complex motility, pushing contents in two directions (a two-component system): the cloaca (excreting as cecal drop) and the ileum (providing bacteria (for bacterial proliferation and colonization)) involved in the bird’s biological homeostasis [197,203,204,205]. The cecum with its high level of diversity maintains intestinal microbial stability in responding to various stressors [206] and determines colonization resistance against invading pathogens [207]. As the bird’s primary fermentative organ, the cecum possesses higher levels of DNA replicative viability than feces [208]. A balanced cecal microbiota diversity and composition have been used as an indicator of growth and health in poultry [209,210,211]. However, unlike mammals, in a commercial production setting, microbial contact is completely interrupted between domesticated parents and chicks. Various technologies have been developed for the modification of gut microbiota diversity and composition and related functions, including CMT, in chickens.

The effects of early-life CMT on the development of the gut microbiota in recipient chickens with long-lasting effects have been previously investigated. Franco et al. reported that broiler chicks (recipients) that received cecal contents from organic hens or industry-raised broilers (donors) by oral application on day 1 had distinctly colonized bacterial microbiota profiles, which was similar to the cecal microbiota profiles of the donors, respectively [212]. The differences between the recipient broilers had been maintained from day 7 to day 42 (the end of this study). The results indicate that transferred microbiota can persistently colonize the newly hatched broilers. In addition, early intervention with cecal fermentation broth from donor broilers (180 days old) regulates the colonization and development of gut microbial function in newly hatched broiler chicks (recipients), increasing beneficial bacteria and the concentration of short-chain fatty acids (SCFAs), while reducing the abundance of pathogenic bacteria [213]. In another study, cecal contents collected from ISA Brown chickens or hens (donors) at 1, 3, 16, 28, and 42 weeks of age were orally applied to newly hatched broiler chicks (recipients) [214]. Its results showed that the cecal proteome of recipient chicks was correlated to the composition of the donors’ microbiome following a single inoculation on the day of hatch, with a long-lasting effect, up to 45 days of age (an entire broiler production period). Taken together, early inoculation with cecal microbiota represents a novel method for modulating the host microbiome to improve production and reduce susceptibility to infection in chickens. 

In the current studies, CMT from the divergently selected inbred donor lines has been evidenced functionally to reduce or inhibit the stress response and related aggression and damage pecking in recipient chickens of a commercial strain. These findings further support the theory that the exhibition of injurious behaviors is a stress-induced neuropsychological disorder in chickens, which is comparable to human psychopathological disorders [215,216]. Stress-associated gut dysbiosis and low-grade chronic inflammation are common traits of these disorders. For group-living chickens as well as other social animals, individuals share microbes and interact around environments and resources, by which the gut microbiota may have considerable consequences for host social interactions, such as the social ranking of individual animals [217,218]. For laying hens, like other social animals, the development of injurious behaviors may therefore be a phenotypic behavioral consequence of an imbalanced gut microbiota composition and related dysregulation of the communication between the gut and brain [204]. Birds with a higher propensity to perform injurious behaviors have distinct microbiota profiles compared to their non-pecking counterparts [27,112]. Similarly, the microbiota differing between the selected inbred lines (line 6_3_ vs. line 7_2_) exhibit distinct phenotypes [112], and CMT may be a method with the potential to control and replicate the role of the gut microbial community after a single passage of transplanted cecal content. This hypothesis will be tested in upcoming studies.

Major microbiota colonization of the intestine occurs in post-hatched chicks. CMT in early life (day-old chicks) may have great protective effects against stress-induced physiological and behavioral changes [219,220]. The current study showed that recipient chickens (6_3_-CMT compared to 7_2_-CMT) had different levels of aggression and related damaging behaviors, which was correlated with the degree of injurious behaviors of donors [123]. The early postnatal period is a vital window for birds as well as mammals to be colonized with the microbiome [213], whereby early-life CMT profoundly influences brain development and intestinal microbiota composition and diversity [221] with a long-lasting impact on gut–brain neural circuit development and its responses to stressful episodes [222,223]. However, inconsistent results of CMT-induced intestinal microbiota modulation have been reported across studies. Early-life homologous (within line) microbiota transplantation (a pooled donor’s ileum, ceca, and colon contents) increases activation in both selected high- and low-FP recipients, with limited effects on their microbiota composition, stress response, and FP [28]. It is still unclear how FP arises as a consequence of dysregulated communication between the gut and the brain. A recent study also reported that gut microbial composition (from the digesta and mucosa of the ileum and cecum) and predicted functions were not associated with FP and antagonistic behavior in laying hens [33]. Therefore, given the inconsistent results, there is a critical need to further identify the biofunctions of cecal microbiota in controlling injurious behaviors in laying hens via CMT from the divergently selected non-aggressive and aggressive lines. Taken together, the obtained results may potentially influence the common procedures used in controlling aggression and related injurious behaviors in chickens as well as other species of farm animals, such as the dehorning of calves in beef and dairy operations [224,225] and teeth clipping or tail docking in swine operations [226,227]. Our work may also have implications for human medicine, providing information for developing next-generation psychobiotics [228,229] and impacting human mental health; currently, 1 in 6 U.S. youth aged 6–17 and 1 in 5 U.S. adults experience mental health disorders each year [230].

## 4. Conclusions and Perspectives

The current results show that differences in behavior, serotonergic activity, stress response, innate immunity, and cecal microbiota populations between the two divergently selected inbred genetic lines (donors, line 6_3_ vs. line 7_2_) can be transferred to other chicken lines (recipients) at an early age (day-old in this study) with long-lasting effects on growth, behavior, and biological functions. The data suggest that the CMT effects are independent of genetic differences between the donors and recipients. The outcomes provide new insights into understanding the underlying mechanisms of the MGB and MGI axes in regulating abnormal behaviors and offer a tractable strategy for reducing social stress and stress-associated injurious behaviors and improving welfare in egg-laying chickens. The roles of individual cecal bacterial members (as the optimal next-generation psychobiotics), the released bioactive factors (as the next-generation agents), and the related biological processes underlying social stress and injurious behaviors in chickens will be examined in the following studies.

## Figures and Tables

**Table 1:** (**A**) Serotonergic metabolism in the raphe nucleus; (**B**) peripheral serotonin, tryptophan, corticosterone, and heterophil/lymphocyte ratios; and (**C**) peripheral immune parameters between the two divergently selected inbred chicken lines 6_3_ and 7_2_.

(A)					
Treatment	5-HT (ng/g)	5-HIAA (ng/g)	5-HT/5-HIAA	TRP	
Line 6_3_	512.6 ^a^	151.8	3.2 ^b^	1183.8 ^a^	
Line 7_2_	352.7 ^b^	168.9	4.9 ^a^	963.2 ^b^	
SEM	8.2	12.9	0.2	22.4	
*p*-value	0.01	0.62	0.04	0.08	
(**B**)					
**Treatment**	**5-HT** **(ng/g)**	**TRP** **(ng/g)**	**CORT** **(ng/mL)**	**H/L ratio**	
Line 6_3_	61.38	171.52 ^a^	8.44 ^b^	0.16 ^b^	
Line 7_2_	59.46	121.42 ^b^	9.75 ^a^	0.50 ^a^	
SEM	3.79	15.37	1.51	0.04	
*p*-value	0.73	0.03	0.05	<0.0001	
(**C**)					
**Treatment**	**IgG** **(mg/mL)**	**IL-6** **(pg/mL)**	**IL-2** **(pg/mL)**	**IL-10** **(pg/mL)**	**TNF-α** **(ng/mL)**
Line 6_3_	12.0	28.14	60.09	9.37	36.65 ^A^
Line 7_2_	12.9	27.56	71.65	13.13	30.73 ^B^
SEM	0.73	1.63	12.8	1.64	2.37
*p*-value	0.54	0.81	0.54	0.12	0.09

Values are least-squares means ± SEM, *n* = 7. ^a,b^ indicate significant differences (*p* ≤ 0.05), ^A, B^ indicate trend differences (0.05 < *p* ≤ 0.10). Abbreviations: 5-HT, serotonin; 5-HIAA, 5-hydroxuindoleacetic acid; CORT, corticosterone; H/L, heterophil-to-lymphocyte ratio; IgG, immunoglobulin G; IL, interleukin; TNF, tumor necrosis factor; TRP, tryptophan [112].

**Table 2:** Effects of cecal microbiota transplantation on body weight, relative organ weight, stress parameters (H/L ratio, corticosterone), and sexual hormone (testosterone) of recipient roosters in week 16.

Measures		Treatment		SEM	*p*-Value
	CTRL	7_2_-CMT	6_3_-CMT		
Body weight	1642.5	1738.6	1711.3	34.3	0.426
Adrenal gland ^1^	4.181 ^AB^	4.762 ^A^	3.306 ^B^	0.420	0.090
H/L ratio	0.327 ^ab^	0.367 ^a^	0.243 ^b^	0.029	0.024
Corticosterone (ng/mL)	4.235	4.678	3.697	0.900	0.789
Testosterone (ng/mL)	1.423	1.132	1.744	0.277	0.345

Values are least-squares means ± SEM, *n* = 7. ^a,b^ indicate significant differences (*p* ≤ 0.05), ^A,B^ indicate trend differences (0.05 < *p* ≤ 0.10). ^1^ Adrenal gland = absolute adrenal gland weight (g)/body weight (kg). Abbreviations: 6_3_-CMT, chickens with cecal bacterial solution of donor line 6_3_; 7_2_-CMT, chickens with cecal bacterial solution of donor line 7_2_; CTRL, control; H/L ratio, heterophil-to-lymphocyte ratio [106].

**Table 3:** Effects of cecal microbiota transplantation on levels of plasma natural IgG concentrations, and pro- (IL-6 and TNF-α) and anti-inflammatory cytokines (IL-10) of recipient roosters in week 5 and week 16.

Treatment	IgG (mg/mL)	IL-6 (pg/mL)	TNF-α (pg/mL)	IL-10 (pg/mL)
Week 5				
CTRL	5.197	38.532	22.846	42.569
7_2_-CMT	5.412	37.109	26.495	33.259
6_3_-CMT	5.245	32.903	26.211	37.503
SEM	0.624	2.014	2.597	5.254
*p*-value	0.565	0.118	0.293	0.499
Week 16				
CTRL	15.032 ^ab^	43.128 ^AB^	16.660	27.467 ^ab^
7_2_-CMT	17.993 ^a^	47.523 ^A^	21.706	26.928 ^b^
6_3_-CMT	13.716 ^b^	38.597 ^B^	16.161	33.835 ^a^
SEM	1.176	3.294	1.896	1.997
*p*-value	0.046	0.070	0.107	0.045

Values are least-squares means ± SEM, n = 7. ^a,b^ indicate significant differences (*p* ≤ 0.05), ^A,B^ show trend differences (0.05 < *p* ≤ 0.10). Abbreviations: 6_3_-CMT, chickens with cecal bacterial solution of donor line 6_3_; 7_2_-CMT, chickens with cecal bacterial solution of donor line 7_2_; CTRL, control; IL, interleukin; TNF-α, tumor necrosis factor alpha [106].

**Table 4:** Effects of cecal microbiota transplantation on mucosal sIgA concentrations and splenic relative mRNA abundance of pro- (IL-6 and TNF-α) and anti-inflammatory cytokines (IL-10) of recipient roosters in week 5 and week 16.

Treatment	sIgA (mg/g)	Relative mRNA Abundance		
		IL-6	TNF-α	IL-10
Week 5				
CTRL	2.167 ^ab^	0.806	0.905	0.396
7_2_-CMT	1.757 ^b^	0.763	1.378	0.461
6_3_-CMT	3.473 ^a^	0.673	1.280	0.258
SEM	0.440	0.141	0.175	0.153
*p*-value	0.045	0.796	0.296	0.456
Week 16				
CTRL	6.433	1.133 ^AB^	2.390 ^AB^	0.879
7_2_-CMT	7.989	1.694 ^A^	2.741 ^A^	0.739
6_3_-CMT	9.914	0.832 ^B^	2.217 ^B^	0.816
SEM	1.369	0.263	0.149	0.266
*p*-value	0.249	0.080	0.065	0.722

Values are least-squares means ± SEM, n = 7. ^a,b^ indicate significant differences (*p* ≤ 0.05), ^A,B^ show trend differences (0.05 < *p* ≤ 0.10). Abbreviations: 6_3_-CMT, chickens with cecal bacterial solution of donor line 6_3_; 7_2_-CMT, chickens with cecal bacterial solution of donor line 7_2_; CTRL, control; IL, interleukin; sIgA, secretory immunoglobulin A; TNF-α, tumor necrosis factor alpha [106].

**Table 5:** Effects of cecal microbiota transplantation on MAOA mRNA expression, serotonergic activities, dopamine, and norepinephrine in the hypothalamus of recipient chickens in week 5 and week 16.

Treatment	MAOA	5-HT (ng/g)	5-HIAA (ng/g)	5-HIAA/5-HT	Tryptophan (ng/g)	Dopamine (ng/g)	Norepinephrine (ng/g)
Week 5							
6_3_-CMT	1.51	496 ^a^	122.3 ^a^	0.225	1784 ^A^	55.7 ^ab^	394 ^AB^
7_2_-CMT	1.56	388 ^b^	86.7 ^b^	0.231	1532 ^AB^	44.5 ^b^	324 ^B^
CTRL	1.45	482 ^ab^	108.3 ^ab^	0.225	1454 ^B^	70.6 ^a^	494 ^A^
SEM	0.11	29.4	6.9	0.014	103.6	6.6	28.1
*p*-value	0.80	0.04	0.007	0.500	0.09	0.03	0.07
Week 16							
6_3_-CMT	2.52 ^a^	397	59.3	0.164 ^a^	2760	121	540
7_2_-CMT	1.80 ^b^	368	50.9	0.131 ^ab^	2140	121	517
CTRL	1.90 ^ab^	384	42	0.110 ^b^	2480	117	506
SEM	0.05	29.5	5.2	0.012	176	9.6	30.7
*p*-value	0.02	0.80	0.23	0.011	0.06	0.94	0.71

Values are least-squares means ± SEM, *n* = 7. ^a,b^ indicate significant differences (*p* ≤ 0.05), ^A,B^ indicate trend differences (0.05 < *p* ≤ 0.10). Abbreviations: 5-HT, serotonin; 5-HAA, 5-hydroxuindoleacetic acid; 6_3_-CMT, received cecal content solution from 6_3_ donors; 7_2_-CMT, received cecal content solution from 7_2_ donors; CTRL, received saline, control; *MAOA*, monoamine oxidase A [123].

## Supplementary Materials

The following supporting information can be downloaded at https://www.mdpi.com/article/10.3390/microorganisms12030471/s1. Figure S1: The examples of the morphological changes of the villus height and crypt depth in the ileum of recipient chickens at (A) week 5 and (B) week 16. Magnification: × 100. Villus height (VH); Crypt depth (CD).

## Abbreviations

5-HIAA, 5-hydroxuindoleacetic acid; 5-HT, serotonin; 6_3_-CMT, a recipient chicken with cecal content solution of line 6_3_; 7_2_-CMT, a recipient chicken with cecal content solution of line 7_2_; ASV, amplicon sequence variant; BW, body weight; BT, beak trimming; CMT, cecal microbiota transplantation; CD, crypt depth; CDI, *C. difficile* infection; CORT, corticosterone; DA, dopamine; ELISA, enzyme-linked immunosorbent assay; EP, epinephrine; FMT, fecal microbiota transplantation; FP, feather pecking; GIT, gastrointestinal tract; GF, germ free; HPA, hypothalamic–pituitary–adrenal axis; H/L, heterophil/lymphocyte ratio; HPLC, high-performance liquid chromatography; Ig, immunoglobulin; IL, interleukin; KP, kynurenine pathway; MGB, microbiota–gut–brain axis; MGI, microbiota–gut–immune axis; NE, epinephrine; SAM, sympathetic–adrenal–medullary axis; sIgA, secretory IgA; SCFA, short-chain fatty acids; SFP, severe feather pecking; SPF, specific pathogen-free; TLR, toll-like receptor; TNF-α, tumor necrosis factor alpha; TRP, tryptophan; VH, villus height.
