# Supplementary material for: The Impact of Early-Life Cecal Microbiota Transplantation on Social Stress and Injurious Behaviors in Egg-Laying Chickens
Source: Microorganisms. 2024 Feb 26;12(3):471. doi: 10.3390/microorganisms12030471 (PMC10972035; doi:10.3390/microorganisms12030471)

microorganisms

MDPI

Figure S1. The examples of the morphological changes of the villus height and crypt depth in the ileum of recipient chickens at A) week 5 and B) week 16. Magnification: × 100. —— Villus height (VH); - - - Crypt depth (CD).

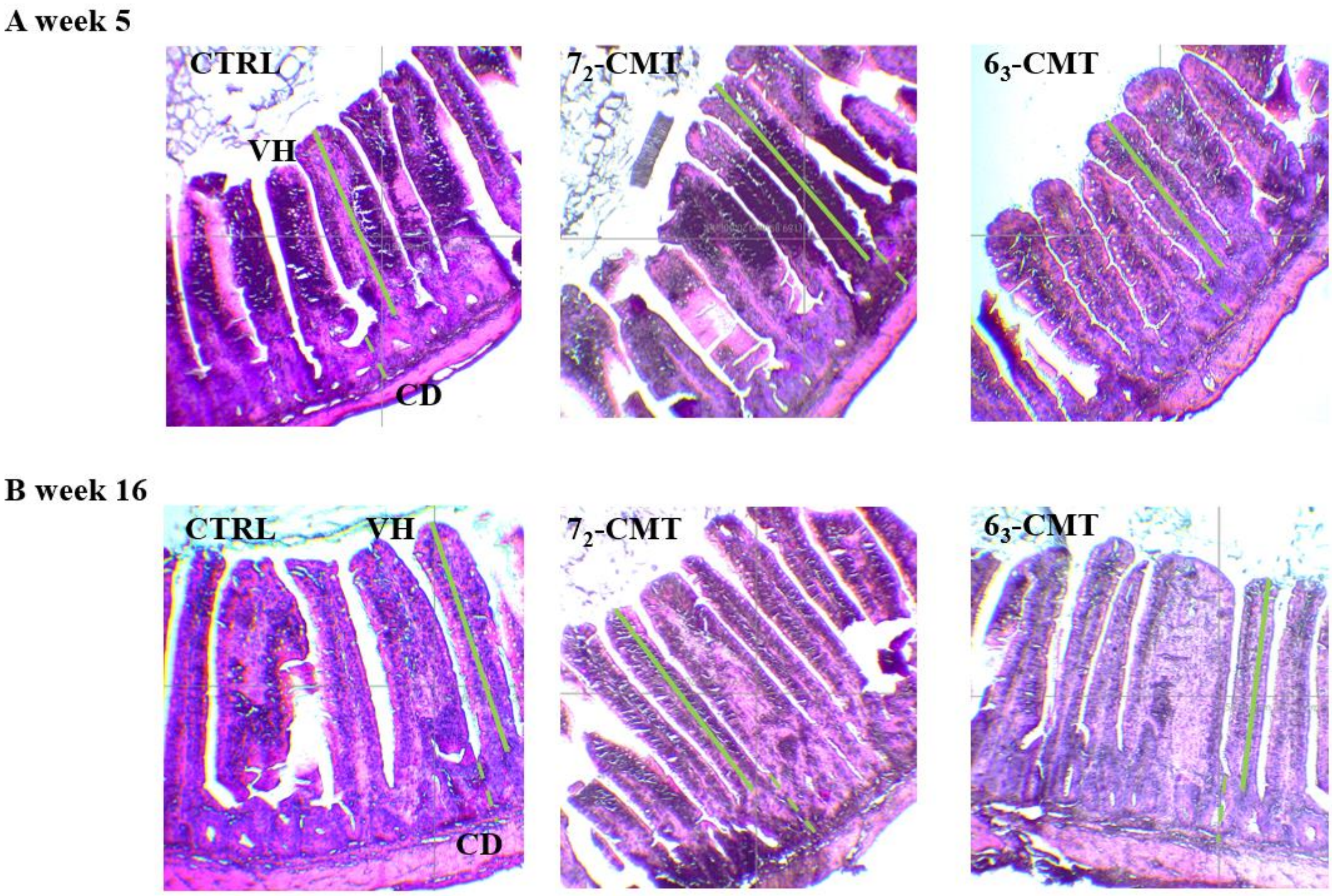

Supplement: Supplementary file 1 [file microorganisms-12-00471-s001.zip › microorganisms-2867064-supplementary.pdf]
